# Supplementary material for: Oportuna Vacuna: A Prospective Study of Vaccine Confidence and Vaccine Uptake in a Low-Income, Spanish-Speaking Rhode Island Population in the Post-Pandemic Era
Source: Vaccines (Basel). 2025 Dec 19;14(1):2. doi: 10.3390/vaccines14010002 (PMC12846445; doi:10.3390/vaccines14010002)
Supplement: Supplementary file 1 [file vaccines-14-00002-s001.zip › Supplement A - Clínica Esperanza Partnership Form_12.18.2025.pdf]

Supplement A:
Partnership Form (English)

Clínica Esperanza Partnership Form

You are being asked to partner with Clinica Esperanza/Hope Clinic (CEHC) in a program to improve the care we offer and measure how that care improves the health of our patients who will eventually become insured. We ask all our patients to take part in this program. Participation in this program is voluntary, and you will have access to CEHC services even if you refuse to participate. Please read this form carefully and ask any questions that you may have before signing this form.

What the program is about: CEHC would like to engage all of our patients in a partnership with the clinic to improve their health. We will work with you to make sure that you have all of the regular checkups, vaccines, and tests that you need to improve your health. If you have medical problems that can be addressed by modifying your diet and changing your exercise habits, we will work with you to make those changes. After you transfer care to another provider we will look into your insurance claims data. The information will be used to help us understand whether your use of CEHC services impact your use of health services through other service providers, such as emergency rooms, hospital admissions and other specialty visits. This will help us determine whether our clinic and our lifestyle modification programs save money for the state of Rhode Island and reduce the cost of healthcare, while also improving the health of the community. As part of this effort, CEHC staff will track the services you receive from the clinic and access data from your future medical provider and/or our data partners when you transfer care. This form asks for your consent to access information maintained by your future medical provider and/or the following data partners:

- Executive Office of Health and Human Services (EOHHS)
- CurrentCare

What we will ask you to do: We ask all clinic patients with chronic diseases to have a follow up with a provider and a one-on-one conversation with a Navegante four times a year. In addition, we ask you to commit to attending (and completing) one group class (Vida Sana, or the Diabetes Prevention Program) or a series of one-on-one conversations with a Navegante for each year that you are an active patient. This allows us to ensure we are offering the highest quality care possible and track how the care we provide improves the health of our patients.

Your information is always confidential. Your information will only be shared with your future medical provider. The purpose of this is so that the medical provider can look up your medical information after you have gained health insurance or transferred care and assess whether the care provided by our clinic improved your health. CEHC will have permission to receive data about you from your future medical provider or our data partners for a period of five years from the date of signature. To do this, we will share information such as your name, birth date or other personal identifier. The data will be secure and de-identified without any personal information.

We will combine your service use information with information from hundreds of other CEHC patients when we analyze data. Your name will never be included in any reports or analysis.

Supplement A:
Partnership Form (English)

CLÍNICA ESPERANZA
HOPE CLINIC

If you have questions: Please ask any staff member questions you have now. If you have questions later, you may contact info@aplacetobehealthy.org or at 401-347-9093 or the Medical Director, Dr. Annie De Groot. You will be given a copy of this form to keep for your records.

Agreement to Partner: I have read the above information and have received answers to any questions I asked. I agree to become a partner with CEHC.

____
Printed Name (Legal)

____
Signature Date
