# Supplementary material for: Oportuna Vacuna: A Prospective Study of Vaccine Confidence and Vaccine Uptake in a Low-Income, Spanish-Speaking Rhode Island Population in the Post-Pandemic Era
Source: Vaccines (Basel). 2025 Dec 19;14(1):2. doi: 10.3390/vaccines14010002 (PMC12846445; doi:10.3390/vaccines14010002)
Supplement: Supplementary file 1 [file vaccines-14-00002-s001.zip › Supplement B - Oportuna Vacuna Patient Consent Form (English).pdf]

Consent Form and Information Sheet for Participants

Title of the study: ***Oportuna Vacuna: A Prospective Study of Vaccine Confidence in a Low Income, Spanish Speaking Population in Providence Rhode Island: Will Increasing Community Health Worker and Healthcare Provider Familiarity with Vaccines and Vaccine Schedules Improve Patient Vaccination Rates?***

Name of Institution: Clínica Esperanza/Hope Clinic
Principal Investigator: Dr Anne De Groot 401-272-2123

Objectives of the study
In response to decreased vaccination rates, Clínica Esperanza/Hope Clinic (CEHC) has launched a research study to examine knowledge, practices and confidence relating to vaccine uptake among healthcare workers [HCW] (medical providers and Community Health Workers [CHW]) and patients as well as the role of vaccine confidence in decisions to vaccinate in a cross-sectional survey at a low-income community clinic serving the Latinx population in Providence. We hope to determine whether improving HCW knowledge, practices and confidence will improve vaccine uptake and the decision to vaccinate at this community-based clinic serving 4,000 low-income Latinx adults.

Why participate in this study?
We will assess confidence in vaccines by surveying patients and healthcare staff from CEHC. By answering the survey questions, you will help CEHC learn about people's knowledge, attitudes, and confidence in vaccines and to help identify the best vaccination strategies that meet the needs and expectations of the community. You will also help CEHC evaluate if training workshops for healthcare workers has an impact on vaccine confidence.

What will you be asked to do?
If you agree to participate in this study, you will answer a series of questions about vaccines [routine vaccines for children, Human papillomavirus (HPV)/cervical cancer, COVID-19] as well as questions about your behaviors and knowledge about vaccine-prevented diseases. Your answers will be collected anonymously by the study staff. At any time, you can withdraw your consent and leave the study. The surveys will be completed before and after the educational interventions. Each survey will take approximately 30 minutes to complete.

What are the risks and benefits of your participation?
The risks associated with your participation in this study include loss of privacy and/or loss of confidentiality, fatigue or boredom. You do not have to answer all questions. You are encouraged to notify the surveyor or study staff if a question makes you uncomfortable.

You will not directly benefit from your participation in the study. Your answers may help to draw conclusions about the acceptance of vaccines in your community.

Confidentiality
Study records will be maintained as confidential as possible. All study records will be kept in password protected files in password protected computers. Any paper documentation will be locked in the clinic safe. Your name will not be assigned to the survey. Your healthcare provider will assign a code to your survey, and the link between your name and the code will be kept securely in a locked safe at your CEHC for up to 5 years. CEHC will only have access to your answers and not to your identity or your medical records. Regulatory authorities, including Salus Institutional Review Board (IRB), may access your study records only to ensure that confidentiality is respected.

Salus IRB
Study #22257
Approved 01/24/2023

Are there any financial implications?
There is no cost to you to participate in this study.

What are your rights as a research participant?
Participation in this study is voluntary. It is your alternative not to participate. Your choice to answer or not to answer the survey will in no way affect your normal care at your health center. If you decide not to participate or to stop your participation, there will be no penalty or loss of benefits to which you are otherwise entitled. You will continue to receive your standard medical care at CEHC.

You can ask any questions you want to the study staff. For any additional information about the study, to report a problem, or if you feel you may have been harmed by participating, or to offer input, you can contact the Principal Investigator, Dr. Anne De Groot at 401-272-2123. You can change your mind at any time.
To leave the study, please contact Dr. De Groot. If you wish to discuss your rights as a study participant with an additional member of our staff, you may contact Jackie Medrano, Project Manager at 401-537-8478.
This study has been reviewed by an Institutional Review Board (IRB) in the United States, Salus IRB. You can call them if you have questions you do not want to discuss with the people listed above, or if you have any questions, concerns, or complaints about your rights as a research participant or regarding this research study. You can contact them using the information below:

Salus IRB
2111 West Braker Lane, Suite 100
Austin, TX 78758
Phone: 1-800-472-3241 between 8:00 AM and 5:00 PM Central Time
Email: subject@salusirb.com

If you would like additional information about your rights, research in general, or IRBs, you may visit www.salusirb.com.

Reference Salus Study: #22257.

Salus IRB
Study #22257
Approved 01/24/2023

Declaration of consent to participate in the study

Title of the study: Oportuna Vacuna: ***A Prospective Study of Vaccine Confidence in a Low Income, Spanish Speaking Population in Providence Rhode Island: Will Increasing Community Health Worker and Healthcare Provider Familiarity with Vaccines and Vaccine Schedules Improve Patient Vaccination Rates?***

The purpose of this research study, the procedures to be followed, and the risks and benefits were explained to me. I was allowed to ask questions, and my questions were answered to my satisfaction.

I was told to contact the Principal Investigator, Dr. Anne De Groot if I have more questions, to discuss problems, concerns or suggestions related to the research, to get information or to offer feedback on the research.

I have read this consent form and agree to participate in this study. My withdrawal from the study at any time is allowed and will not change my medical follow-up in any way. I have been told that I will get a copy of this consent form when it is signed. I do not give up any of my legal rights by signing this consent form.

I voluntarily agree to take part in this study. If you do not want to participate, do not sign this form.

____

Printed first and last name of the participant

Signature

For consent obtained orally: "X" or fingerprint

____

DATE (dd/mm/year)

____

Printed name and signature of the person explaining and obtaining consent

____

DATE (dd/mm/year)

Salus IRB
Study #22257
Approved 01/24/2023

OBTAINING ORAL CONSENT

In the case of obtaining oral consent, a witness outside the family and the study must attest to the obtaining of consent. Please sign below.

I was present through the consent process. I am not part of the study team or the participant's family. I certify that the above information was explained, all questions were answered, and that consent was given freely, without constraint.

____

Printed name and surname and signature of the witness

____

Date

Salus IRB
Study #22257
Approved 01/24/2023
