# Supplementary material for: Oportuna Vacuna: A Prospective Study of Vaccine Confidence and Vaccine Uptake in a Low-Income, Spanish-Speaking Rhode Island Population in the Post-Pandemic Era
Source: Vaccines (Basel). 2025 Dec 19;14(1):2. doi: 10.3390/vaccines14010002 (PMC12846445; doi:10.3390/vaccines14010002)
Supplement: Supplementary file 1 [file vaccines-14-00002-s001.zip › Supplement C - Figures and Tables_12.18.2025.pdf]

Table S1: Age and Gender of Chart Review Subjects

	CR1 (N=816)			CR2 (N=709)		
**Age Range**	**Female**	**Male**	**Transgender**	**Female**	**Male**	**Transgender**
18–27	38	40	0	27	35	0
28–37	58	75	1	50	64	1
38–47	112	107	0	103	94	0
48–57	95	118	0	82	105	0
58–67	57	60	0	48	52	0
68–77	28	19	0	23	18	0
78–87	6	2	0	6	1	0
Total	*394*	*421*	*1*	*339*	*369*	*1*
Percent	*48.3%*	*51.6%*	*0.1%*	*52.0%*	*52.0%*	*0.1%*

Table S1. Summarizes the ages and genders of subjects from the first and second chart reviews. Ages were categorized into consecutive 10-year bands (18–27, 28–37, 38–47, etc.) for analysis. Genders were self-identified.

Table S2 – Chart Review: Vaccine Uptake

Percent of Vaccines Received *(if eligible)*	CR1	CR2
0%	306	177
1–9%	0	0
10–19%	251	197
20–29%	143	123
30–39%	15	46
40–49%	58	60
50–59%	26	59
60–69%	3	11
70–79%	7	19
80–89%	5	14
90–99%	0	0
100%	2	3

Table S2. Patients are grouped by their relative vaccine uptake (percentage of recommended vaccines completed) and the number of patients in each uptake group between Chart Review 1 and Chart Review 2 is compared.

Figure S1 – Staff Survey: Question 9

Q9: *For each line, check the disease(s) that are prevented by each vaccine*	**IPV**		**Varicella**		**PCV20**		**Tdap**		**MenB**		**BCG**		**HepB**		**MMR**		**Hib**		**HPV**		**Zoster**		**I don't know**	
	Pre	Post	Pre	Post	Pre	Post	Pre	Post	Pre	Post	Pre	Post	Pre	Post	Pre	Post	Pre	Post	Pre	Post	Pre	Post	Pre	Post
Cervical cancer																			4	4			1	
Diphtheria			1		1			4															2	
Haemophilus Influenzae type B						1							1		1		1	3					2	
Hepatitis B		1											4	3									1	
Measles															3	4							2	
Meningitis									0	0					2			1					2	1
Mumps															3	4							2	
Pneumococcal disease					3	4																	1	
Poliomyelitis	1	4																					3	
Rubella				1											3	3							1	
Shingles			1																		0	3	4	1
Tetanus								4																
Tuberculosis	1										1	4											2	
Whooping cough					2			4															2	

Figure S1. Medical providers' responses to survey question 9 before (Pre) and after (Post) receiving training regarding 2023 CDC guidelines is recorded in this figure. Green = correct answer, and red = incorrect answer. Yellow = "I don't know".
