# Supplementary material for: Oportuna Vacuna: A Prospective Study of Vaccine Confidence and Vaccine Uptake in a Low-Income, Spanish-Speaking Rhode Island Population in the Post-Pandemic Era
Source: Vaccines (Basel). 2025 Dec 19;14(1):2. doi: 10.3390/vaccines14010002 (PMC12846445; doi:10.3390/vaccines14010002)
Supplement: Supplementary file 1 [file vaccines-14-00002-s001.zip › Supplement D_OV Patient Survey - English_PDF.pdf]

Survey to be given to patients enrolled in the *Oportuna Vacuna* study

This survey can be offered to all adult patients presenting to CEHC's Main Clinic or NHS in English or Spanish.

The CEHC staff who give the questionnaire to patients must have received training on CEHC's vaccination information campaign, the objective of the *Oportuna Vacuna* program, how to give this questionnaire and how to keep the documentation.

During the questionnaire, the notes for the interviewers are indicated on a grey background. <u>They should NOT be read to patients.</u>

Before we begin, please verify that participants have been informed of the purpose of the survey and have signed the informed consent form. Indicate the date and patient ID below. The center codes are shown below.

This questionnaire is in six parts:

1. General information.. p1
2. Issues relating to vaccination in general p2
3. EPI questions.. p3
4. Questions relating to HPV and HPV vaccine p3-4
5. Questions relating to COVID-19 .... p4-5
6. Questions related to new COVID-19 vaccines p6

Date: |__|__|- |__|__| -|__|__|__|__| **Identifier:**
DD MM YYYY Study Center Identifier

Health Center:
☐ **CEHC (60 Valley St.)**
- ☐ Provider visit (PV)
- ☐ One-on-one (1-1)

☐ **NHS (85 Eagle St.)**
- ☐ Vida Sana (VS)
- ☐ Vida Pura (VP)
- ☐ Health Fair (HF)
- ☐ One-on-one (1-1)

Gender:
☐ Male
☐ Female
☐ Nonbinary
☐ Other: ____________

Age: ________________

Do you have children:
☐ Yes
☐ No

Level of education completed
☐ Elementary School
☐ High School
☐ College
☐ Graduate School
☐ None

How often do you attend a religious service?
☐ Weekly
☐ Monthly
☐ Only for religious holidays
☐ Never

Immigration Status
☐ Naturalized citizen
☐ Refugee/Asylee
☐ Lawful permanent resident (LPR)
☐ Undocumented
☐ Other (please specify)

Identifier: |__|__| - |__|__|__| - |__|__|__|

2. QUESTIONS FOR VACCINES IN GENERAL

For each line, check the box that best reflects your feelings about vaccines in general:

	Completely Disagree	Disagree	No opinion	Agree	Completely Agree
1. I think vaccines are safe					
2. I think vaccines are important for children					
3. I think vaccines are effective					

4. Have you tried to get information about vaccines in the last 30 days? ☐ Yes ☐ No
If yes: Which vaccines?
☐ Vaccines in general
☐ HPV vaccines
☐ COVID-19 vaccines
☐ Other (specify opposite)

Allow the patient respond and check all the answers that apply.

5. Would you like to know more about vaccines? ☐ Yes ☐ No
If yes: Which vaccines?
☐ Vaccines in general
☐ HPV vaccines
☐ COVID-19 vaccines
☐ Other (specify opposite)

Let the patient answer and check all the answers that apply.
If the patient answers yes, please give information about CEHC's vaccine offerings, other information sources, or advise to contact their doctor.

6. Indicate how often you **follow the recommendations** of the people/entities below regarding vaccination.

	Always	Often	Sometimes	Never
The healthcare team of CEHC (Navegantes, doctors, nurses)				
Traditional healers				
Government and health authorities				
Local representatives (city councilperson, mayor, governor, etc.)				
Religious leaders				
Celebrities (actors, singers, athletes)				
My father				
My mother				
Other family members				
My friends/neighbors				
Other (specify):				

7. Indicate if you have been exposed to the following, and if they have influenced your confidence in vaccines.

Check one answer per line.

	I have been exposed and my confidence in			Not exposed
	Lessened	Unchanged	Increased	
Discussion with CHWs on vaccination				
Discussion with a HCP				
Discussion in a class (Vida Sana, Vida Pura)				
Social media post				
A poster				
Other (specify):				

Identifier: |__|__| - |__|__|__| - |__|__|__|

3. QUESTIONS RELATED TO CHILDHOOD VACCINATIONS – for participants with children

8.	Have your children been vaccinated under the expanded program on immunization?<br>If not, what are the reasons?<br>***(Check all the reasons that apply)***	☐ Yes, all ☐ Yes, some ☐ No ☐ I have no children<br>☐ My children are not at risk<br>☐ My children are not exposed<br>☐ I prefer my children to have the disease rather than the vaccine<br>☐ I'm worried about side effects / it's not safe<br>☐ I am against the vaccine in general<br>☐ Vaccines are not effective<br>☐ It's against my religion<br>☐ It takes too much time and money<br>☐ Other (specify opposite)
9.	Do you know anyone who has been severely affected or has died from one of the diseases covered by routine vaccinations?<br>***Summarize the diseases and check yes if the patient identifies*** one or more disease(s)<br>Diphtheria, tetanus, pertussis, hepatitis B, yellow fever, measles, Haemophilus influenza type B (Hib), poliomyelitis, rotavirus, tuberculosis, meningitis, pneumococcal disease.	☐Yes ☐ No
10.	Have you had to cancel vaccination appointments because of the COVID-19 pandemic?	☐Yes ☐ No ☐ I don't know
11.	Have you had to cancel vaccination appointments for reasons other than the COVID-19 pandemic in 2020, 2021, and 2022?	☐Yes ☐ No ☐ I don't know
12.	Were you able to replace these cancelled appointments?	☐Yes ☐ No ☐ I don't know

4. HPV ISSUES

13.	Do you know about cervical cancer?	☐Yes ☐ No
14.	Have you ever heard of the HPV vaccine?	☐Yes ☐ No
15.	Do you think there is a link between the HPV vaccine and cervical cancer prevention?	☐Yes ☐ No<br>☐ I don't know
16.	Have you ever heard of the cervical cancer vaccine?	☐Yes ☐ No
17.	Would you agree to vaccinate your children against HPV / cervical cancer?	☐Yes ☐ No<br>☐ I don't know
18.	This vaccine protects against a sexually transmitted disease that causes cervical cancer. It is used in many countries and is available in the United States Does this information change your answer to the previous question?	☐Yes ☐ No<br>☐ I already knew

Identifier: |__|__| - |__|__|__|__| - |__|__|__|__|

19. Let's say you are the director of the CDC. Who should the vaccine be given to?

To babies	☐Yes	☐ No	☐ I don't know
To boys before marriage	☐Yes	☐ No	☐ I don't know
To girls before marriage	☐Yes	☐ No	☐ I don't know
To adult men	☐Yes	☐ No	☐ I don't know
To adult women	☐Yes	☐ No	☐ I don't know

5. COVID-19 ISSUES

20.	Do you know about COVID-19?	☐Yes ☐ No
21.	Do you know the cause of Covid-19?<br>***(The response must include infection with a virus)***	☐Yes ☐ No
22.	Do you think this is a major risk to the health of the community?	☐Yes ☐ No ☐ I don't know
23.	Do you think COVID-19 still exists in Providence, Rhode Island ? If not, what are the reasons?<br>**(Check *all the reasons that apply*)**	☐Yes ☐ No ☐ I don't know<br>☐ I never believed it<br>☐ We do not hear more about it<br>☐ Not enough cases<br>☐ Other (specify opposite)
24.	Do you think the COVID-19 virus survives in hot countries?	☐Yes ☐ No ☐ I don't know
25.	Do you think that the official figures relating to COVID-19 (number of sick and dead) reflect reality?	☐Yes ☐ No ☐ I don't know
26.	Do you fear a new wave of COVID-19 contamination?	☐Yes ☐ No ☐ I don't know
27.	Do you think COVID-19 can be a serious illness?	☐Yes ☐ No ☐ I don't know
28.	Who do you think COVID-19 affects **more often:**	
	Kids	☐Yes ☐ No ☐ I don't know
	The elderly	☐Yes ☐ No ☐ I don't know
	People who are overweight or obese	☐Yes ☐ No ☐ I don't know
	The poor	☐Yes ☐ No ☐ I don't know
	Rich people	☐Yes ☐ No ☐ I don't know
	The sickly	☐Yes ☐ No ☐ I don't know
29.	Do you know someone who has been sick with the symptoms of COVID-19 but has not been tested?<br>***(Please explain the symptoms: difficulty breathing, flu-like illness, loss of smell or taste)***	☐ Yes ☐ No
30.	Do you know anyone who has been slightly ill with COVID-19?	☐ Yes ☐ No
31.	Do you know someone who has been seriously ill or has died as a result of Covid-19?<br>Yes, how many? Choose the nearest number	☐ Yes ☐ No<br>☐ 1 ☐ 5 ☐ 10 ☐ 20 or more
32.	Has your healthcare staff ever discussed COVID-19 prevention methods with you?	☐ Yes ☐ No ☐I don't remember
33.	Currently, do you practice barrier gestures?	☐Yes ☐ No ☐ I don't know
34.	Do all the adults in your home have masks?	☐Yes ☐ No ☐ I don't know
35.	Have you ever been tested for COVID-19?	☐Yes ☐ No ☐ I don't know

Identifier: |__|__| - |__|__|__|__| - |__|__|__|__|

36. How do you greet your friends?

With an embrace	☐ Yes	☐ No
Shaking hands	☐ Yes	☐ No
By touching the point	☐ Yes	☐ No
By touching the elbow	☐ Yes	☐ No
Contactless	☐ Yes	☐ No

37. For each line, check the box that best reflects your habits **OVER THE PAST MONTH:**

	Not concerned	Never	Sometimes	Often	Always
I wash my hands when I get home					
I wear a mask outside the house					
I practice physical distancing					

38. Have you heard any rumors about COVID-19 and COVID-19 vaccines? If so, which ones and specify whether you believe in them or not.

(Let the patient answer and check the answers that apply)

	I've heard about ...			Never heard
	I believe in it	I don't believe it	It's possible	
The **virus** was created by a foreign government				
The **virus** was created by Bill Gates				
Vaccine contains microchips				
Vaccine contains magnets / makes my arm magnetic				
The vaccine will change my DNA				
The vaccine will give me COVID-19				
The vaccine will change me into a zombie				
All COVID-19 vaccines cause blood clots				
Once vaccinated, I am protected against COVID-19				
Other:				

Identifier: |__|__| - |__|__|__|__| - |__|__|__|__|

6. ISSUES RELATED TO THE NEW VACCINES AGAINST COVID-19

39.	Have you been vaccinated against COVID-19?<br>If yes: Date of first injection: ______/20_____<br>Month Year<br>Have you completed the vaccination?<br>***(Please specify that vaccination Sinovac, SPUTNIK, Pfizer, Moderna or Astrazeneca is done in two doses, Johnson and Johnson in one dose)***<br>Were you sick after the injection? If yes, specify symptoms:	☐ Yes ☐ No<br><br>☐ Yes ☐ No<br><br>☐ Yes ☐ No
40.	Do you know anyone who has been vaccinated against COVID-19?<br>If so, was this person sick afterwards?	☐ Yes ☐ No<br>☐ Yes ☐ No ☐ I don't know
41.	If you have not been vaccinated, would you agree to be vaccinated against COVID-19?<br>If you do not agree to be vaccinated, or if you are hesitant, what are the reasons?<br>***(See all the reasons that apply and add the other reasons below)***<br>Would you agree to be vaccinated in exchange of money?<br>If you agree, or if you have already been vaccinated, what are the reasons?<br>***(See all the reasons that apply and add the other reasons below)***	☐ Yes ☐ No ☐ I don't know<br>☐ I don't think it will be prudent<br>☐ I'm not at risk enough to get covid-19<br>☐ I am worried about side effects<br>☐ I am against the vaccine in general<br>☐ I don't think it will work<br>☐ It's against my religion<br>☐ I'm sick / I don't feel well<br>☐ I want a different vaccine from the one offered to me<br>☐ Other (specify)<br>☐ Yes ☐ No ☐ I don't know<br>☐ Protect myself from the disease<br>☐ Protect those around me (family / friends)<br>☐ Protecting seniors in my family<br>☐ Protecting children<br>☐ I follow the instructions<br>☐ Other (specify)
42.	Who would you advise to receive the COVID-19 vaccine?	☐ To the elderly<br>☐ To my family<br>☐ To children<br>☐ To my friends / colleagues / neighbors<br>☐ I do not recommend it<br>☐ Other (specify)

End of questionnaire: Please thank the patient for their time and contribution.

Identifier: |__|__| - |__|__|__| - |__|__|__|
